# Morphological Control of Single-Concave Elastomeric Colloid through Cross-Linking and Osmotic Pressure Variations for Chemical Delivery

**DOI:** 10.1021/acsami.5c03818

**Published:** 2025-04-02

**Authors:** Yi-Chen Ho, Ting-Yu Xu, Chieh-Yun Juan, Yi-Shan Lai, Yu-Fang Lai, Pei-Chieh Tseng, Han-Yu Hsueh

**Affiliations:** † Department of Materials Science and Engineering, National Chung Hsing University, Taichung 40227, Taiwan, Republic of China; ‡ Innovation and Development Center of Sustainable Agriculture, National Chung Hsing University, Taichung 40227, Taiwan, Republic of China

**Keywords:** colloid, buckling, osmotic pressure, concave, elastomer

## Abstract

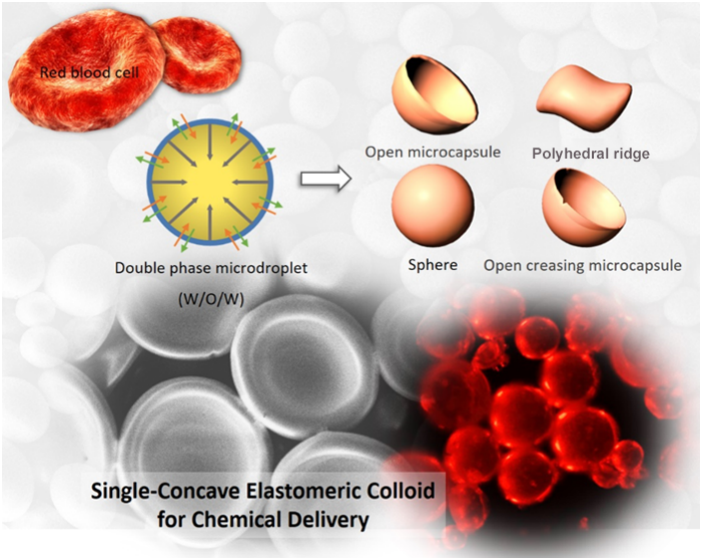

In this paper, we propose a convenient and simple method for preparing elastomeric buckled spherical shells through double-emulsion solvent evaporation. In this method, polydimethylsiloxane (PDMS) is added to a water-in-oil-in-water emulsion system and stirred. As the solvent evaporates and the colloid solidifies, the aqueous phase inside formed cavity is squeezed out because of cross-linking stress, or this phase permeates the sphere, leading to loss of the internal water phase and the formation of a single-concave, bowl-shaped shell (i.e., a shell with buckling deformation). We could control the morphology of the produced colloidal particles by varying the concentrations of the permeant and cross-linking agent and the molecular weight of the permeant. Moreover, we propose a mechanism explaining the structural changes occurring during the double-emulsion polymerization process, focusing on cross-linking forces and osmotic pressure. Through leveraging of the shells’ reversible swelling properties in solvents, the prepared buckled PDMS shells absorbed Nile red molecules into their cavity, which caused their expansion and restoration. Immersing these shells in ethanol resulted in release of the Nile red molecules. Thus, buckled PDMS shells prepared through the proposed method have potential for application in environmental sensing and drug delivery systems.

## Introduction

The shape is a crucial structural characteristic of particles in polymer colloids and significantly affects their practical functions and overall properties. In this context, a nonspherical shape can be regarded as an inherent functional property of colloidal particles,^1^ which have unique optical, rheological, and mechanical properties. Polymer particles with different shapes—such as raspberry-like,^2^ dumbbell-like,^3^ mushroom-like,^4^ ellipsoidal,^5^ disk-like,^6^ cylindrical,^7^ and golf-ball-like^8^ shapes—have attracted considerable academic and industrial interest. However, the creation of spheres with surface concavities, such as buckled spherical shells, remains particularly challenging.

Buckled spherical shells are spherical shells that have experienced sudden collapse because of a compressive load. At the onset of buckling, the external pressure surpasses a critical threshold, which results in the thin-walled elastic structure becoming unstable and the formation of single or multilateral indentations on the surface.^9,10^ Numerous factors influence the buckling paths and postbuckling structures of homogeneous spherical shells, including axisymmetry, shear stress, locally compressive stress, viscoelasticity, and osmosis.^1,11−14^ Extensive simulation-based research has been conducted on stable configurations of structures susceptible to buckling as well as the thresholds required for these states to transition between different structures.^15^ Munglani et al. conducted a series of simulations on the collapse morphology of anisotropic spherical shells and obtained three findings. First, the critical buckling pressure of these shells is affected by the Young’s modulus, Poisson ratio, shell radius, and shell thickness. Second, certain regions in anisotropic shells are predisposed to deform inward, resulting in breaking of symmetry and a reduction in critical pressure. Finally, three distinct postbuckling regimes exist for anisotropic shells.^16^ Osmotic pressure plays a vital role in the invagination process. Osmotic deflation occurs when a concentration gradient exists between the inner and outer sides of a semipermeable capsule membrane; the deformation continues until the concentration reaches equilibrium. Kierfeld et al. developed a quantitative theory that describes the relationships between osmotic pressure, elastic modulus, and collapse capsule volume to categorize the shift from a spherical shape to an axisymmetric buckled shape under osmotic pressure, mechanical pressure, and volume control.^17^

The mechanism of formation and fabrication of buckled spherical shells has been extensively investigated. However, most research on their formation mechanism has been based on theoretical simulations.^17−23^ For example, Meng et al. studied the morphology evolution of drying soft matter droplets using a pseudodynamic analysis. They demonstrated that the elastic properties of the surface gel layer control both early stage instabilities and later morphological changes. Through a quasi-equilibrium energy minimization approach, they explored the transitions in droplet shape, providing key insights into the fabrication of microparticles with desired morphologies.^18^ Knoche et al. theoretically explained the complete sequence of shapes involved in the formation of deflated spherical shells. As the volume of a spherical shell decreases, it initially remains spherical; it then undergoes classical buckling instability, where an axisymmetric dimple appears; finally, it loses its axisymmetry as wrinkles develop near the dimple edge during a secondary buckling transition.^19^ Few researchers have investigated the aforementioned process through a combination of theoretical and experimental methods. Quilliet et al. prepared oil-filled thin shells through emulsion templating. These shells exhibited buckling in mixtures of water and ethanol because their core dissolved in such mixtures. This phenomenon led to the generation of conformations with a single axisymmetric or polygonal depression, depending on the shells’ geometric features.^20^ Most experimental research on nonspherical polymer particles has focused on rigid polymer systems (i.e., nonelastomeric materials), such as polystyrene,^24,25^ divinylbenzene,^26^ poly(methyl methacrylate),^27,28^ polyelectrolyte,^29^ and block copolymers.^30−32^ For example, Zhang et al. reported a simple method for the synthesis of monodisperse red-blood-cell-shaped polystyrene particles. Their method involves using divinylbenzene as a cross-linker in ethanol or an ethanol–water mixture, with the concentration of divinylbenzene, the feeding mode of the cross-linker, and the ethanol/water mass ratio being varied as appropriate.^24^ The nonspherical structures formed in nonelastomeric material systems lack reversible structural characteristics. In contrast, elastomeric buckled spherical shells may exhibit reversible structural characteristics; however, limited experimental research has explored this aspect. As mentioned earlier, most studies on elastomeric buckled spherical shells are based on simulation results.

In the present study, we developed a fast, convenient, and simple method for preparing elastomeric buckled spherical shells through double-emulsion solvent evaporation. In this method, polydimethylsiloxane (PDMS) is placed in a water-in-oil-in-water (W_1_/O/W_2_) emulsion system and stirred. As the solvent evaporates and the colloid solidifies, the aqueous phase within the cavity is squeezed out due to cross-linking stress, or this phase permeates in and out of the spheres, leading to the loss of the internal water phase and resulting in the formation of a single-concave, bowl-shaped shell (i.e., a shell with buckling deformation). We were able to control the morphology of the colloidal particles by varying the concentration and molecular weight of the permeant, as well as the concentration of the cross-linking agent. Moreover, we identified a mechanism that reasonably explains the structural changes occurring during the double-emulsion polymerization process, based on the cross-linking driving force and osmotic pressure. In an application utilizing their reversible swelling property in solvents, the prepared buckled PDMS spherical shells absorbed Nile Red molecules into their cavity, causing shell expansion and subsequent restoration. When these shells were immersed in ethanol, they released the absorbed Nile Red molecules. Thus, PDMS spherical shells prepared through the proposed method have potential applications in environmental sensing and drug delivery systems.

## Results and Discussion

### Roles of Osmotic Pressure and Cross-Linked Stress in the Formation of Buckled Elastomeric Spherical Structures

**Scheme 1:**
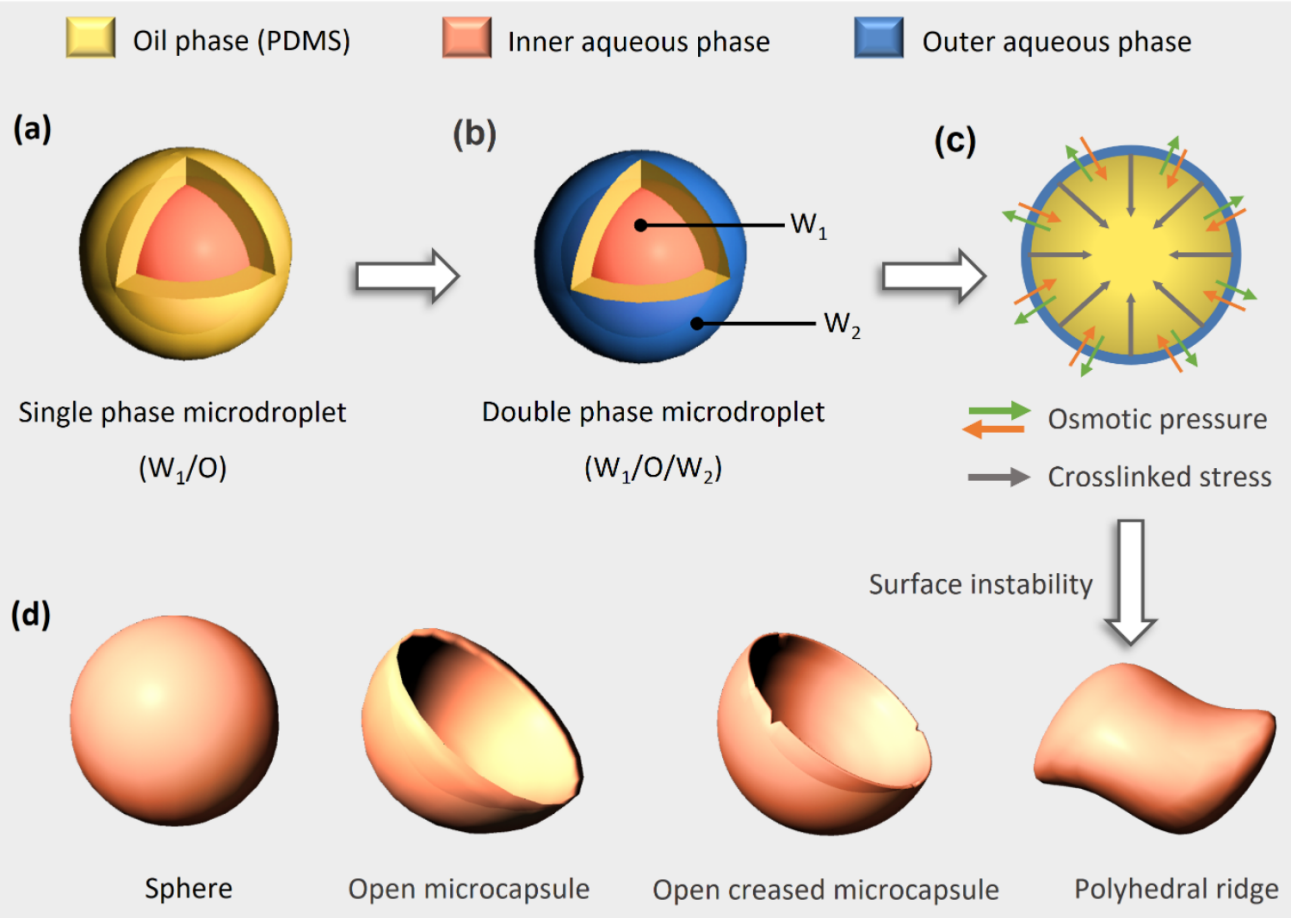
Schematic of the Formation of Buckled PDMS Spherical Structures after Surface Instability of Multiphase Emulsified Microdroplets: (a) Generation of Water-in-Oil (W_1_/O) Single-Phase Microdroplets [Yellow, Orange, and Blue Represent Liquid PDMS/Chloroform Solution (O), the Internal PVA Solution (W_1_), and the External PVA Solution (W_2_), Respectively]; (b) Generation of Water-in-Oil-in-Water (W_1_/O/W_2_) Double-Phase Microdroplets; (c) Formation of Osmotic Pressure (Red and Green Arrows) and Cross-Linked Stress (Gray Arrows) during the Heating Process; and (d) Obtained Buckled PDMS Structures (i.e., Structures with Polyhedral Ridge, Spherical, Open Microcapsule, and Open Creased Microcapsule Geometries)

The double-emulsion solvent evaporation method was used to fabricate buckled microspheres by inducing surface instability. First, liquid PDMS precursor was added to mixtures containing varying weight ratios of a cross-linking agent and chloroform. The mixtures were stirred until no visible two-phase separation was observed, forming an oil phase (O). Next, the obtained mixtures were agitated and added to a poly(vinyl alcohol) (PVA) solution, which served as the internal aqueous phase (W_1_), thus producing water-in-oil (W_1_/O) single-phase microdroplets (Scheme 1a). The solution was stirred consistently, causing the emulsion to turn milky. Then, the well-mixed W_1_/O emulsions were poured into PVA and water mixtures, which acted as the external aqueous phase (W_2_), resulting in uniform water-in-oil-in-water (W_1_/O/W_2_) emulsions, or dual-phase microdroplets (Scheme 1b). The solutions were then heated to form buckled PDMS spherical structures. During heating, two forces acted on the system: osmotic pressure between the internal and external aqueous phases, and cross-linking stress on the spherical shell (Scheme 1c). These forces controlled the interfacial tension of the elastomer and determined the formation of dual-phase microdroplets with various buckled spherical structures. After the PVA solution and unreacted PDMS precursor were removed through centrifugation and drying, buckled PDMS structures with geometries such as polyhedral ridges, spherical shapes, open microcapsules, and open creased microcapsules were obtained. These structures have potential applications in drug delivery (Scheme 1d).

**Figure 1:**
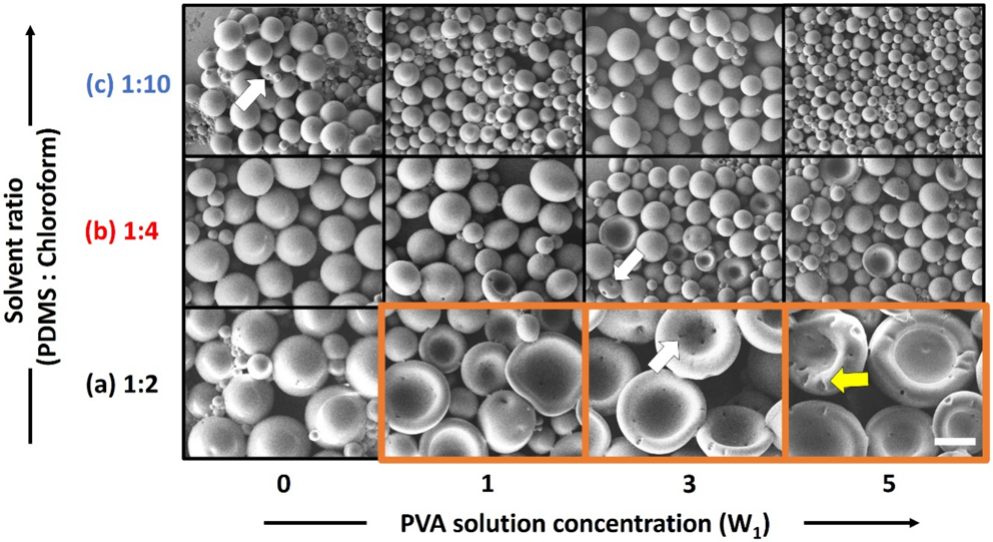
SEM images of buckled spherical structures fabricated using PDMS/chloroform solutions and aqueous PVA solutions with different concentrations: structures produced under PDMS:chloroform ratios of (a) 1:2, (b) 1:4, and (c) 1:10. The concentration of W_1_ ranged from 0 to 5 wt %, and the scale bar represents 100 μm.

**Figure 2:**
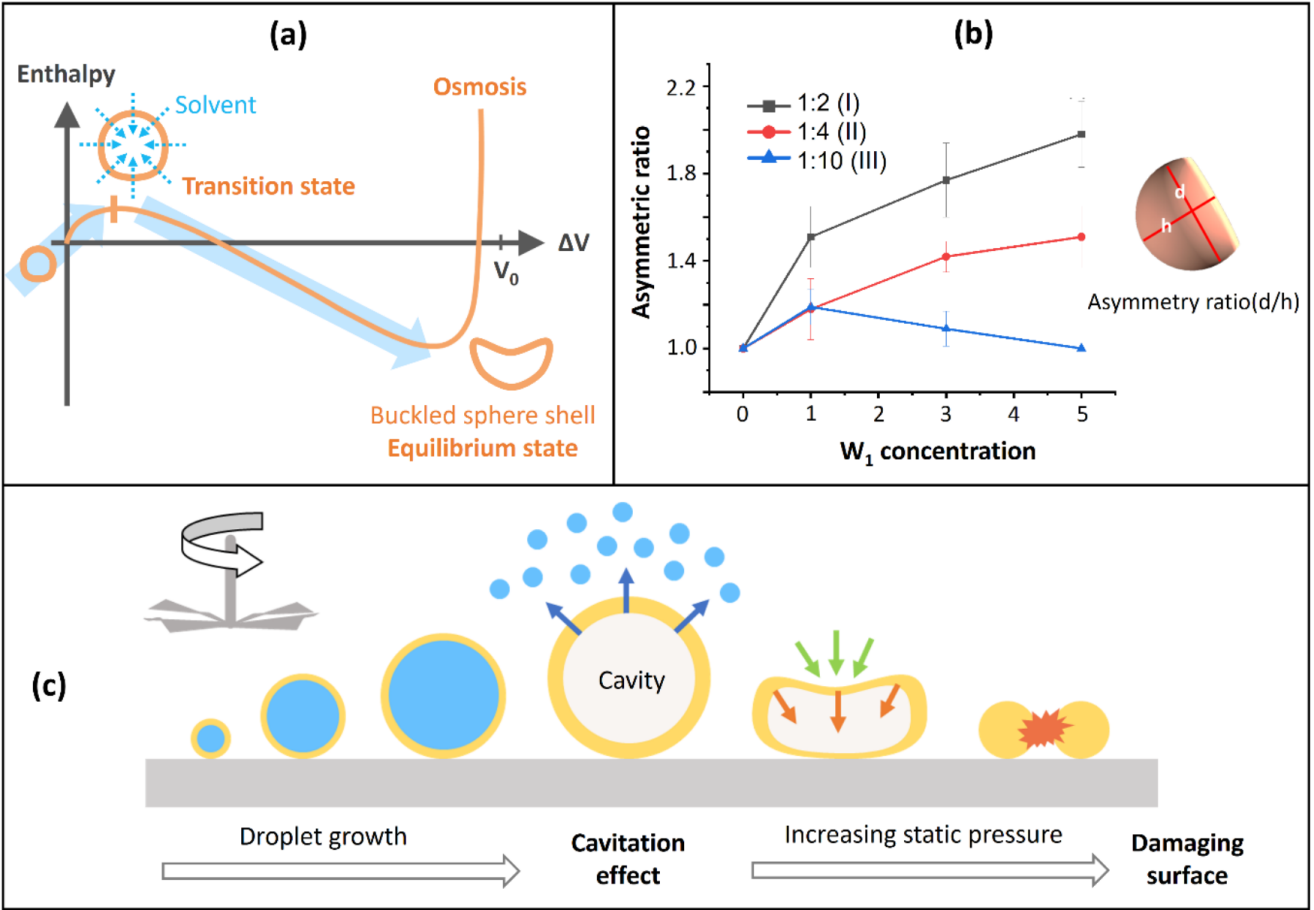
(a) Effects of the concentration of the internal aqueous phase (W_1_) and the diameter of the prepared PDMS spherical shells on the osmotic free energy and morphology of the produced spherical micelles. (b) Variation in the asymmetry ratio of the formed micellar structures with the W_1_ concentration. Inset: definition of the asymmetry ratio. (c) Cavitation process of multiphase emulsified microdroplets.

Figure 1 displays electron microscopy images of buckled spherical structures produced using different concentrations of PDMS/chloroform solution (O, oil phase) and aqueous PVA solution (W_1_). The concentration of the external aqueous phase (W_2_), which consisted of a PVA and water mixture, was fixed as 1 wt % in this study. The microdroplets retained their solid spherical structures and did not exhibit cavities or collapse when no aqueous PVA solution (W_1_: 0 wt %) was present in them (Figure 1). This stability was primarily attributed to the low surface energy of the PDMS-based oil droplets, which made water penetration into the oil phase energetically unfavorable. Disruption of the oil–water interface would increase the interfacial free energy, creating a significant energy barrier to water infiltration. Moreover, the oil phase (PDMS in chloroform) exhibited a minimal capacity to dissolve water, rendering the osmotic driving force negligible. The small volume of PDMS micelles, in the absence of an internal aqueous phase, further limited the accommodation of additional water molecules. Additionally, the cross-linked structure of PDMS acted as a physical barrier, effectively preventing water molecules from penetrating the oil phase. However, addition of PVA solution to the oil phase resulted in the spherical structures deforming and collapsing. The degree of collapse depended on the internal aqueous phase (W_1_), which was affected by the osmotic free energy of the internal and external solutions.^33^ The osmotic free energy is expressed as follows:
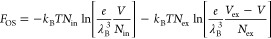
1

where *k*_B_ is the Boltzmann constant; *T* is the temperature of the solutions;  is the thermal de Broglie wavelength, with *h* and *m* representing the Planck constant and particle mass, respectively; *N*_in_ and *N*_ex_ are the numbers of osmotically active particles inside and outside the shell, respectively; *V* is the internal volume of the shell; and *V*_ex_ – *V* is the external volume of the shell. Note that the morphological change of the microcapsule arose from the competition between two types of energy: osmotic free energy between W_1_ and W_2_, and elastic potential energy of PDMS shell. When the PVA concentration in W_1_ increased, the internal osmotic pressure rose, driving the external solvent (W_2_) to flow into the internal phase (W_1_), which caused an expansion in the capsule volume (*V*). An increase in the W_1_ concentration causes an increase in *N*_in_ in the internal aqueous phase. When *N*_in_ increases, the first term of eq 1 decreases, as does the osmotic free energy. This osmotic pressure reduced the concentration difference between the internal and external phases, theoretically stabilizing the capsule. However, as the capsule expanded, the elastic shell (PDMS) experienced increasing stress, leading to the accumulation of elastic potential energy. When the volume reached a transition state (Figure 2a),^17,21^ the elastic stress in the shell approached its stability limit. To minimize the total energy, the system released localized stress through a morphological change. This resulted in the formation of a dimpled region (i.e., buckled PDMS spherical structures), which effectively reduced the total elastic potential energy and stabilized the microcapsule in a single-dimpled shape, approaching a flattened form. This shape represented a balance between osmotic free energy and elastic potential energy.

Figure 1a indicates that as the concentration of the internal aqueous phase (W_1_) was increased, the number of buckled spherical shells formed in the system was greater than the number of intact spherical shells formed (highlighted by the orange lines in Figure 1a). This finding confirmed the aforementioned statement. In addition, the spherical shells formed through solvent evaporation, which had a size of around 10 μm (wall thickness of the bowl-like structure), had a rough surface and pores or buckles (white arrow in Figure 1). When a solvent was used to produce microspheres, the solvent became trapped inside the microspheres during the fabrication process. As the solvent evaporated and the microspheres shrunk because of capillary force, the chimney effect occurred, which resulted in the formation of pore-like structures. Not all solvents cause microspheres to appear porous. The solvent type, solvent concentration, and microsphere generation conditions influence whether microspheres appear porous. For example, the chloroform evaporated quickly when the PDMS:chloroform ratio was 1:2 and 1:4 in the present study. Under this condition, PDMS did not have repairability because it did not undergo swelling; thus, the PDMS exhibited larger pore-like structures at the aforementioned solvent ratios than when the solvent ratio was 1:10. Moreover, creases formed on the edges of the produced buckled spherical shells because of surface instability arising from the difference between the concentrations of the internal and external aqueous phases as well as variation in the tension at the liquid–liquid interface (yellow arrow in Figure 1). This tension is expressed as follows:
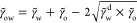
2

The tension at the liquid–liquid interface depended on the surface tension of the two liquids. For the PVA solution, solute and water molecules can migrate freely and exert attractive forces on their neighboring molecules. The H-bonds between water molecules result in a strong affinity between the molecules themselves, contributing to the liquid’s surface tension. Moreover, this bond causes PVA to have a high-free-energy state, which results in PVA being driven toward the liquid–liquid interface between the PVA solution and the oil phase. If excessive PVA accumulates at the surface, it increases the surface tension locally. However, this accumulation of PVA at the interface can lead to a reduction in the overall surface tension of the PVA solution, as the PVA molecules replace water molecules at the surface and lower the energy required to maintain the interface.^34^ If the concentration of the external aqueous phase is fixed, the difference between the surface tension of the two liquids is greater when the concentration of W_1_ is higher. A larger tension difference causes the liquid–liquid interface to become increasingly unstable,^35^ which results in creases being formed on the prepared spherical shells to reduce the free energy.

Figure 2b displays the relationship between the W_1_ concentration (0, 1, 3, and 5 wt %) and the asymmetry ratio of the formed micellar structures. The asymmetry ratio is obtained by dividing the initial particle diameter *d* by the postdeformation particle diameter *h*. A ratio closer to 1 corresponds to the formed morphology being closer to spherical. As depicted in Figure 2b, for the PDMS:chloroform ratio of 1:2 (line I), the asymmetry ratio increased with the W_1_ concentration, which is in agreement with the images shown in Figure 1a. However, for a solvent ratio of 1:10 (line III), an increase in the W_1_ concentration caused a decrease in the asymmetry ratio. This result was attributable to the presence of air bubbles within the PDMS spheres; these bubbles were generated during stirring. Table S1 presents the measured diameters and corresponding morphological values of the buckled PDMS microcapsules under different fabrication conditions. As chloroform evaporated and the spheres shrank because of cross-linking, the pressure within the spheres increased, leading to rupturing of the air bubbles within them. This phenomenon, which is known as cavitation, resulted in fragmentation of the material (Figure 2c). Air bubbles tended to accumulate more readily in a sparser structure. In addition to the cavitation effect, the PVA concentration (W_1_) was also a critical factor influencing the PDMS sphere size. Higher PVA concentrations in W_1_ can reduce the interfacial tension between W_1_ (PVA solution) and the oil phase (PDMS in chloroform). This reduction in interfacial tension may contribute to the formation of smaller droplets during the emulsification process. Furthermore, higher PVA concentrations can accelerate the stabilization of the emulsions, which, in turn, facilitates faster solvent evaporation. This accelerated evaporation might further promote the size reduction of the PDMS spheres. During the process, PDMS spheres with an original size of 80 μm were transformed into buckled PDMS spheres with a size of approximately 25 μm (Figure 1b). Small spheres with high specific surface tension and low surface pressure could withstand external pressure and resist deformation. Therefore, spherical shell buckling was not observed when the PDMS:chloroform ratio was 1:10 (line III). The curve of the asymmetry ratio versus W_1_ concentration had similar shapes for PDMS:chloroform ratios of 1:4 (line II) and 1:2 (line I); however, the curve was considerably higher for the PDMS:chloroform ratio of 1:2. Spherical structures with various sizes and buckled spherical shells coexisted in the system when the PDMS:chloroform ratio was 1:4. However, only buckled spherical shells existed in the system when the PDMS:chloroform ratio was 1:2. Therefore, to ensure the generation of reversible microspheres for subsequent chemical adsorption and release applications, a PDMS:chloroform solvent ratio of 1:2 was employed in the following experiments.

### Factors Affecting the Formation of Buckled Spherical Shells

**Figure 3:**
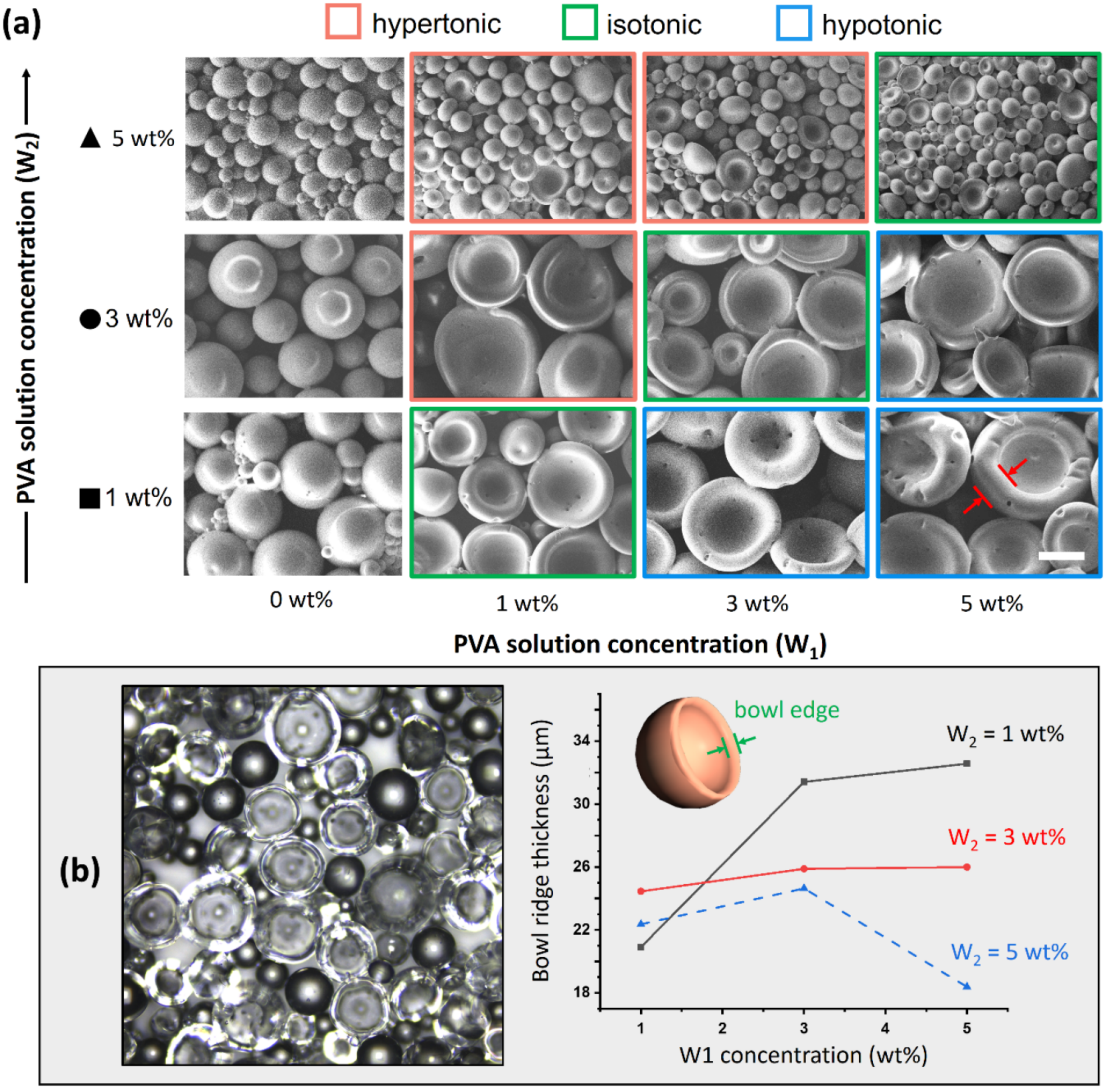
(a) SEM images of buckled spherical structures fabricated under different W_1_ and W_2_ concentrations. The scale bar represents 100 μm. (b) Confocal microscopy image of bowl-like buckled spherical structures (left section) and relationships of the thickness of the bowl edge with the W_1_ and W_2_ concentrations (right section).

A PDMS microsphere functions as a small-scale osmosis system when in solution. Therefore, we investigated how the structure of PDMS microspheres varied with the concentration of the external aqueous PVA phase (W_2_; Figure 3). The microspheres maintained their spherical shape when the W_1_ concentration was 0 wt %. The external aqueous PVA solution provided an osmotic environment and served as a surfactant, allowing the microspheres to disperse uniformly in the solution. In addition, the surfactant reduced the surface tension of the microspheres and stabilized the new surface produced during homogenization (the process of breaking down particles into smaller, more uniform sizes), which resulted in the generation of smaller spheres.^36^ When the W_1_ concentration was 0 wt %, the average microsphere diameter changed from approximately 150 μm to varying sizes with an increase in the W_2_ concentration. Smaller spheres can better resist buckling. Therefore, when the W_1_ and W_2_ concentrations were 5 wt %, 60-μm buckled spherical shells (diameter of the bowl-like structure, not the shell thickness) and 20-μm microspheres formed. According to eq 1, as the W_2_ concentration increases, *N*_ex_ also increases, which leads to a decrease in *F*_os_, thus stabilizing the system. Under these conditions, buckled spherical shells tend to form. The influences of *N*_in_ and *N*_ex_ on the free energy indicate that the system should exhibit a preference for the production of buckled spherical shells because of their lower free energy at higher W_2_ concentrations. However, the morphological transformation illustrated in Figure 3 does not follow the aforementioned theory. This discrepancy occurred because a higher surfactant concentration resulted in the formation of smaller microspheres in the system. This phenomenon caused a decrease in Δ*V* (Δ*V* = *V*_0_ – *V*) and promoted the formation of spherical structures (Figure 2a). Consequently, when the W_2_ concentration was 5 wt %, microspheres were the predominant structure, whereas buckled spherical shells coexisted with microspheres as the W_1_ concentration was increased.

PDMS acts as a semipermeable membrane, providing channels for the permeation of the internal and external aqueous phases. This setup renders each microsphere an independent osmotic system. The structure of PDMS microspheres resembles that of red blood cells in the human body. These microspheres exhibit selective behaviors similar to those of cell membranes, which only permit the passage of solvents, gases, and certain small molecules. Consequently, PVA molecules in the solution cannot penetrate the PDMS through osmosis, whereas water can freely pass through it. The W_2_ solution is classified as a hypertonic solution (highlighted with red lines in Figure 3a), an isotonic solution (highlighted with green lines), or a hypotonic solution (highlighted with blue lines) when its concentration is higher than, equal to, or lower than that of the W_1_ solution, respectively (Figure 3a). Confocal microscopy was employed in this study to determine the thickness of the produced buckled spherical shells. The thickness of the bowl edge (marked by green arrows in Figure 3b) was positively correlated with the W_1_ concentration. When the concentration difference between the sides of the PDMS shell was greater, the osmotic pressure gradient was higher, and a higher number of solvent molecules moved from the lower-concentration solution to the higher-concentration one. When the W_1_ concentration exceeded the W_2_ concentration, water penetrated the produced PDMS microspheres, resulting in thicker microspheres (Figure 3b). However, when the W_2_ concentration was 5 wt %, the produced PDMS microspheres became thinner in a hypertonic solution (blue dotted line in Figure 3b). At this W_2_ concentration, buckled spherical shells coexisted with microspheres, which caused random variation in the thickness of these shells. Higher concentrations of surfactants may lead to the formation of a greater number of smaller microspheres, which can exhibit structural irregularities. Also, the interactions between the microspheres and the buckled spherical shells can result in variations in thickness. When both microspheres and buckled spherical shells coexist, their shapes and sizes may influence each other, contributing to the uneven thickness of the shells.

**Figure 4:**
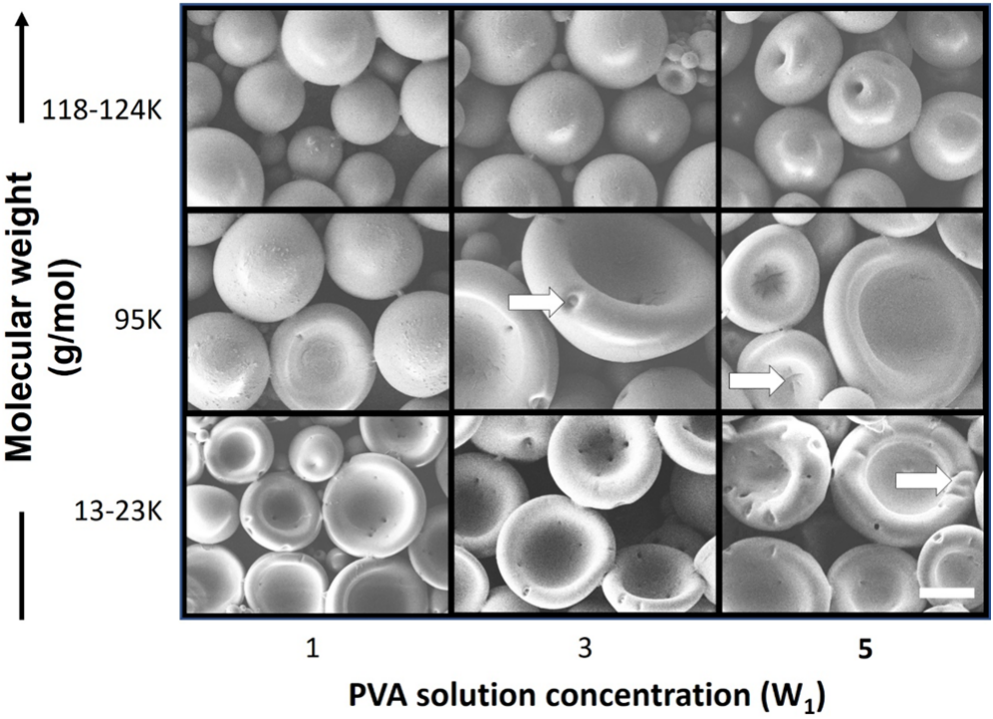
SEM images of PDMS micellar structures fabricated from PVA solutions (W_1_) with different concentrations and different molecular weights of PVA. The scale bar represents 100 μm.

We also investigated the effects of the W_1_ concentration and the molecular weight of PVA on the morphologies of the produced PDMS micellar structures (Figure 4). Research indicates that higher PVA concentration results in lower liquid–liquid interfacial tension.^35^ When the concentration of the PVA solution (W_1_) was increased from 1 to 5 wt %, the difference between the interfacial tension values on the inner and outer sides of the PDMS shells increased, resulting in the formation of bowl-like shells with creased edges (creasing is indicated by white arrows in Figure 4). This phenomenon occurred because the shells had to withstand higher compression force as the difference between the interfacial tension values was increased; thus, cavity deformation occurred. Moreover, a higher molecular weight of PVA resulted in higher surface tension of W_1_,^34^ which caused the aqueous PVA droplets to occupy a greater volume within the PDMS shells. Consequently, obtaining buckled PDMS spherical shells was difficult; a spherical morphology was maintained because PVA with a higher molecular weight has higher mechanical strength to resist the collapse of PDMS spheres. For example, under a W_1_ concentration of 1 wt %, when the molecular weight of PVA was increased from 13,000–23,000 to 118,000–124,000 g/mol, the PDMS morphology changed from buckled spherical shells to intact spherical shells (Figure 4). Therefore, PVA with a molecular weight of 13,000–23,000 g/mol was used as the permeating agent in subsequent experiments to fabricate stable buckled spherical shells.

Rotational speed affects the morphology of PDMS emulsions. To fabricate W_1_/O emulsions, system was operated at a rotational speed ranging from 800 to 1200 rpm (rotational speed range I). Subsequently, W_1_/O/W_2_ emulsions were prepared under a rotational speed ranging from 500 to 1500 rpm (rotational speed range II). At higher rotational speed, the resultant PDMS emulsion was more fragmented because of the resulting stronger shear forces (Figure S1). Specifically, at the highest value in rotational speed range I and lowest value in rotational speed range II (1200 and 500 rpm, respectively), W_1_/O emulsions formed numerous small spheres surrounded by PDMS shells (white arrow in Figure S2), which resulted in the creation of multicore double emulsions. Consequently, buckled PDMS shells did not develop because insufficient space for collapse was available within the PDMS emulsions; polyhedral ridge structures formed instead (highlighted by red lines in Figure S1). The thickness of the buckled PDMS spherical shell could be adjusted by varying the PDMS–chloroform mixing ratio. When the quantity of chloroform was greater, the PDMS was more diluted, which resulted in the formation of thinner buckled spherical shells after surface instability and PDMS cross-linking. However, because more solvent molecules hindered polymer chain cross-linking, a longer cross-linking time was required to produce stable buckled spherical shell structures (highlighted by red lines in Figure S3). The formation mechanisms described in the aforementioned text are also applicable to other elastomeric material systems, such as epoxy resin systems. Figure S4 illustrates various buckled epoxy spherical structures fabricated from PVA solutions with concentrations ranging from 1 to 5 wt %. Similar to the morphological transition observed in the PDMS system, increasing the PVA concentration caused the produced epoxy microdroplets to transition from intact spheres to buckled spherical shells. These buckled shells had a rough and porous surface because of solvent evaporation.

### Demonstration of Reversible Adsorption and Release Behavior

**Figure 5:**
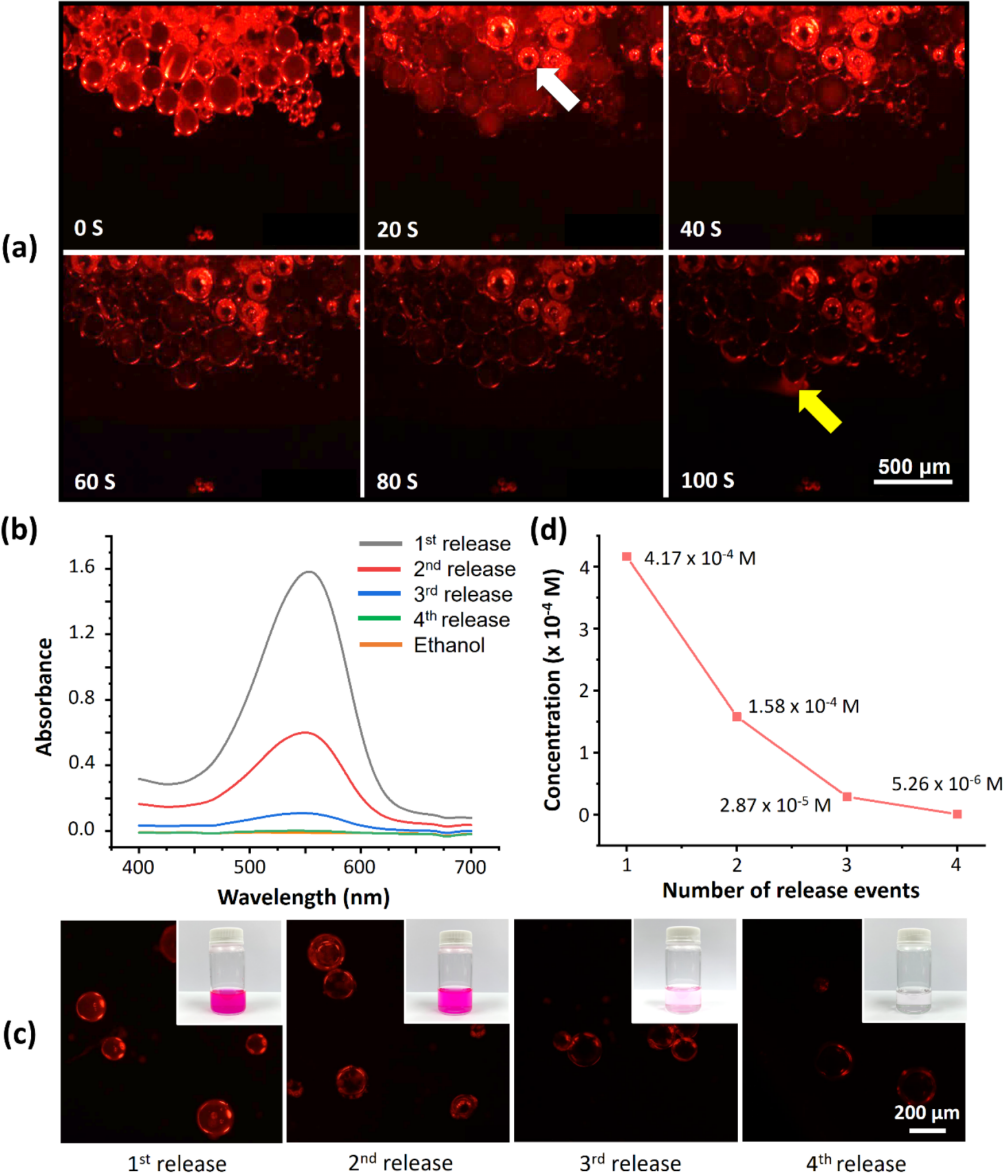
(a) Images obtained through inverted fluorescence microscopy of open elastomeric microcapsules adsorbing and releasing Nile red dye. (b) Ultraviolet–visible absorption spectra of Nile red solutions in ethanol after each release cycle. (c) Images obtained through inverted fluorescence microscopy of the microcapsules after different release cycles. Inset: corresponding optical microscopy images. (d) Calculated residual dye concentrations in the ethanol solution after each release process.

Nile red was used as a probe material to investigate the chemical adsorption and release abilities of the prepared buckled spheres. Initially, open microcapsules without staining were soaked in Nile red solution (10^–3^ M), which resulted in these microcapsules transforming into spheres because of the swelling caused by dye infiltration. Observations conducted using an inverted fluorescence microscope indicated that the prepared spherical structures exhibited vibrant red fluorescence (Figure 5a, 0 s). This fluorescence was caused by the absorption of Nile red dye by the spheres and confirmed that Nile red molecules had successfully infiltrated the inner regions of the capsules; thus, complete spherical structures formed. After the spheres had adsorbed Nile red dye, they were subjected to an evaporation process in air to observe their dye release process. As the evaporation time increased, indentations (indicated by the white arrow) appeared at the center of the red fluorescent spheres. This phenomenon occurred because the evaporation of ethanol resulted in the complete spherical structures transforming back into open microcapsules. The formation of open microcapsules caused a reduction in the encapsulated volume, leading to the extrusion of Nile red molecules from the spheres. This phenomenon was evidenced by the gradual weakening of the fluorescence of the spheres and the appearance of faint fluorescence in the area outside the capsules (indicated by the yellow arrow). This faint fluorescence originated from the Nile red molecules, previously encapsulated within the spheres, that were expelled and dispersed into the surrounding space. Eventually, the spheres returned to a dark state with no fluorescence, indicating that all the Nile red had been released from the spheres (Figure 5a, 20–100 s).

To observe the process of dye chemical release in the system in greater detail, we performed the release process multiple times and measured the fluorescence intensity of the waste solutions. After Nile red dye was released from the spherical capsules, it was collected using ethanol and subjected to ultraviolet–visible spectroscopy. Moreover, the formed open microcapsules were reswollen with ethanol to revert them back into spheres. This process was performed four times, and the dye concentrations in the waste solution were compared (Figure 5b). As expected, with an increase in the number of release cycles, the amount of released dye decreased, resulting in progressively weaker fluorescence intensity. Moreover, the collected Nile red waste solution became increasingly lighter and more transparent (Figure 5c). The aforementioned phenomena indicated that fewer fluorescent molecules remained inside the spheres; thus, fewer molecules were washed out. After the fourth wash, fluorescence spectroscopy and ultraviolet–visible spectroscopy confirmed that the buckled capsules were almost entirely clean and in their original state. Finally, the waste solution became transparent and colorless, and the spheres no longer exhibited any fluorescent response. The residual dye concentration in ethanol after each release process was expressed using the Beer–Lambert equation, which is expressed as follows:^37^
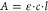
3where *A* is the absorbance, ε is the molar absorptivity, *c* is the concentration of the solute in the solution, and *l* is the path length of light passing through the medium. According to the aforementioned equation, the absorbance is directly proportional to the solute concentration. The *A* value obtained from the peaks with the highest absorbance after each release process is displayed in Figure 5b. The *A* values for the first, second, third, and fourth releases were 1.583, 0.600, 0.109, and 0.002, respectively. Furthermore, ε and *l* were 38,000 L/(mol·cm) and 1.0 cm, respectively. The residual dye concentrations in the ethanol solution after Nile red release were calculated (Figure 5d); these calculations revealed that the residual dye concentrations in the solutions were lower after each release process. As shown in Figure S5, the micelles released 41.7% of the adsorbed dye within the first 100 s, indicating a rapid initial release phase. By 400 s, 60.9% of the dye had been released, with the release rate gradually decreasing after 100–200 s. This trend suggests an initial burst release followed by a more sustained diffusion-controlled phase. This release behavior follows the Higuchi model for diffusion-controlled systems, described by the equation:
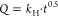
4where *Q* is the cumulative amount of drug released (typically expressed in mg/cm^2^ or M), *k*_H_ is the Higuchi release constant, and *t* is the release time. By fitting the experimental data to the Higuchi model using *Q* vs *t*^0.5^, we estimated the Higuchi release constant as *k*_*H*_ ≈ 3.2 × 10^–5^ M/s^0.5^. The goodness of fit, *R*^2^ ≈ 0.98, suggests that the release behavior aligns well with the Higuchi model, confirming a diffusion-controlled mechanism. Overall, the aforementioned results indicate that elastomeric buckled spheres have potential for application in the pharmaceutical, food, cosmetic, agricultural, and paint industries. Nevertheless, approximately 30% of the dye remained unreleased, likely due to entrapment within the cross-linked PDMS molecular network. Hydrophobic interactions between the dye and the PDMS matrix, along with steric hindrance in the polymeric structure, further restricted diffusion and release. Adjusting the cross-linking density could provide better control over drug release kinetics. Additionally, as PDMS exhibits solvent-induced swelling in certain environments, mild swelling conditions may facilitate further release. Investigating external stimuli, such as ultrasound or pH variations, could be an effective strategy to enhance the release of the residual 30%. While the Higuchi model adequately describes the early stage release behavior, deviations observed at later stages suggest the involvement of additional mechanisms, such as polymer relaxation or molecular entrapment. To better capture the overall release dynamics, we are currently evaluating the applicability of a combined diffusion-relaxation model, such as the Korsmeyer–Peppas equation. These ongoing studies aim to refine the mechanistic understanding of drug release from PDMS-based microcapsules and provide insights into optimizing their design for controlled-release applications. This work is in progress.

## Conclusions

In this study, we developed a fast, convenient, and straightforward method for preparing single-concave spherical elastomer structures via double-emulsion solvent evaporation. In this process, PDMS is dispersed in a W_1_/O/W_2_ emulsion system and stirred. As the solvent evaporates and the colloid solidifies, cross-linking stress expels or permeates the aqueous phase inside the cavity, leading to water loss and the formation of a concave, buckled shell. By adjusting the concentration and molecular weight of the permeant as well as the cross-linking density, we can control the morphological evolution of colloidal particles, generating various structures, including polyhedral ridges, open microcapsules with creases, open microcapsules, and spheres. Furthermore, we propose a mechanism explaining these structural transformations during the double-emulsion polymerization process, driven by cross-linking forces and osmotic pressure. This mechanism allows for predicting the final morphology of PDMS colloidal particles and can be extended to other elastomeric material systems, such as epoxy-based systems.

We also demonstrated that elastomeric buckled PDMS shells can absorb Nile Red molecules into their cavities, expand, and subsequently release the dye upon immersion in ethanol. While most studies on nonspherical particles focus on nonelastomeric polymer systems,^38^ few have investigated elastomeric polymer systems, particularly for chemical release purposes. In nonelastomeric polymer systems (e.g., polystyrene), chemical release typically occurs via capsule rupture or porous diffusion, where pores are introduced through etching or material blending. These microcapsules usually facilitate a one-time release, losing functionality once the drug is discharged. In contrast, our elastomeric PDMS microcapsules offer a more flexible release mechanism, influenced by mechanical stress, osmotic pressure, and cross-linking density, enabling reversible adsorption and release. Additionally, PDMS’s inherent microporosity allows for tunable permeability, controlled by environmental factors such as osmotic pressure and cross-linking density. Owing to their reversible dye absorption/release capabilities and anisotropic geometry, elastomeric buckled PDMS spheres hold potential for applications in drug delivery, imaging contrast agents, insect repellents, surface-enhanced Raman scattering (SERS) sensors, and oil–water separation systems.

## Experimental Section

### Materials

In this study, buckled PDMS microcapsules were prepared using PDMS with a precursor/cross-linker mixing ratio of 5:1 (Sylgard-184, Dow Corning). The cross-linking agent used in this study was methylhydrosiloxane, which is hydrophobic and exhibits negligible solubility in aqueous phases. The epoxy resin used in this study was E-1188TH (Product ID: 0972293523, Fong Yong Chemical Co., Ltd., Taiwan). The solute of the aqueous phase in the double-emulsion method comprised PVA with molecular weight of 13,000–23,000 g/mol (Sigma-Aldrich), 95,000 g/mol (Sigma-Aldrich), and 118,000–124,000 g/mol (First Chemical Works). The concentration of the PVA solution was adjusted from 0 to 5 wt % by appropriately adding deionized water to the solution. Chloroform (Sigma-Aldrich) was selected as the oil-phase solvent because of its excellent solubility in PDMS. Finally, the Nile red used for dye absorption experiments was purchased from Thermo Fisher Scientific, and ethanol solvent was purchased from J.T. Baker.

### Fabrication of Buckled PDMS Microcapsules

PDMS emulsions were prepared through double-emulsion solvent evaporation by using a W_1_/O/W_2_ emulsion system, in which the aqueous phases W_1_ and W_2_ were PVA solutions. The PVA molecules act like surfactants herein. The internal aqueous phase (W_1_) had a concentration of 0, 1, 3, or 5 wt %, and the external aqueous phase (W_2_) had a concentration of 1, 3, or 5 wt %. The oil phase comprised a mixture of PDMS precursor and cross-linker in chloroform, with the weight ratio being 1:2, 1:4, or 1:10. The double-emulsion solution was prepared at a W_2_:O:W_1_ volume ratio of 30:10:3. Initially, the W_1_ solution was added to the oil-phase mixture and stirred at 800–1200 rpm in a sealed container by using a mechanical stirrer (RW20 digital, IKA) for 10 min to prepare water-in-oil (W_1_/O) emulsion. Subsequently, the emulsion was added to the W_2_ solution and subjected to secondary emulsification through mechanical stirring at 500–1500 rpm in a sealed container for 3 h. The resulting double-emulsion solution was then mechanically stirred at 60 °C in an oil bath for 6 h to enable PDMS cross-linking and solidification, after which the chloroform was evaporated. The suspension containing the cross-linked PDMS shells was centrifuged at 8000 rpm for 10 min in a centrifuge (CN-10001, Hsiang Tai). The supernatant was then removed, and deionized water was added to it to redisperse the precipitate. This washing procedure was performed thrice to completely remove unreacted precursors and surfactants.

### Fabrication of Buckled Epoxy Microcapsules

In the fabrication of buckled epoxy microcapsules, W_1_ and W_2_ were again PVA solutions. In this fabrication, the W_1_ concentration was 1, 3, or 5 wt %, and the W_2_ concentration was fixed as 1 wt %. The oil phase was a mixture of epoxy precursor and cross-linker in chloroform, with the weight ratio being 1:2. The double-emulsion solution was prepared at a W_2_:O:W_1_ volume ratio of 30:10:3. The reaction conditions for synthesizing the buckled epoxy microcapsules were similar to those for synthesizing the buckled PDMS microcapsules. First, the W_1_ solution was added to the oil-phase mixture, which was stirred at 1000 rpm in a sealed container by using a mechanical stirrer for 10 min to prepare a water-in-oil emulsion (W_1_/O). This emulsion was then added to W_2_ and subjected to secondary emulsification through mechanical stirring at 1000 rpm in a sealed container for 3 h. The resulting double-emulsion solution was mechanically stirred at 60 °C in an oil bath for 6 h to facilitate cross-linking and solidification of the epoxy precursor, after which the chloroform was evaporated. The suspension containing the cross-linked epoxy shells was centrifuged at 8000 rpm for 10 min. The supernatant was then removed, and deionized water was added to it to redisperse the precipitate. This washing procedure was conducted thrice to completely remove unreacted precursors and surfactants.

### Absorption and Release of Dye

The testing samples used for the absorption and release experiments were prepared under the following conditions: a PDMS-to-chloroform ratio of 1:4, PVA with a molecular weight of 13–23 K, W_1_ at 3 wt %, W_2_ at 1 wt %, and rotation speeds of 1000/500 rpm. First, Nile red was dissolved in ethanol to create 10^–3^ M solution. In the dye absorption process, PDMS open microcapsules whose excess water had been removed through centrifugation were immersed in the prepared Nile red solution and subjected to ultrasonic oscillation for 30 min to enable them to swell and absorb the dye. Subsequently, the suspension was centrifuged at 8000 rpm for 10 min. The solution was then decanted, and deionized water was added to it to redisperse the PDMS microcapsules. This washing step was performed thrice to completely remove any excess Nile red dye. In the dye release process, the Nile red dye was released from the spherical PDMS capsules, collected using ethanol (10 mL), and analyzed through ultraviolet–visible spectroscopy. The open microcapsules were then reswollen with ethanol to revert them back into spheres. This process was conducted four times.

### Characterization

The microstructures of the prepared buckled elastomeric microcapsules were characterized through field-emission scanning electron microscopy (JSM-6700F, JEOL) and laser scanning confocal microscopy (LEXT OLS5000, Olympus). Images of dye absorption and release were captured using an inverted fluorescence microscope (IX73, Olympus) and a hand-held digital microscope (AM4115ZTL, Dino-lite), respectively. Finally, fluorescence spectrum analysis was conducted through ultraviolet–visible spectroscopy (UH5700, HITACHI).

## Supporting Information Available

Electron microscopy images of PDMS micellar structures fabricated under different rotational speeds (Figure S1); Images extracted from a video showing the formation of buckled PDMS shells and polyhedral ridge structures from single-core and multicore double-emulsion solutions (Figure S2); Electron microscopy images of PDMS micellar structures fabricated using PDMS–chloroform solutions with different mixing ratios and cross-linking times (Figure S3); Electron microscopy images of buckled spherical epoxy structures fabricated from PVA solutions (W_1_) with different concentrations (Figure S4); Percent cumulative concentration of Nile Red dye released from PDMS microcapsules into the ethanol surroundings (Figure S5). Table S1 presents the measured diameters and corresponding morphological values of the buckled PDMS microcapsules under different fabrication conditions (PDF)
