# Supplementary material for: Morphological Control of Single-Concave Elastomeric Colloid through Cross-Linking and Osmotic Pressure Variations for Chemical Delivery
Source: ACS Appl Mater Interfaces. 2025 Apr 2;17(15):23414–24. doi: 10.1021/acsami.5c03818 (PMC12012692; doi:10.1021/acsami.5c03818)
Supplement: Supplementary file 1 — am5c03818_si_001.pdf [file am5c03818_si_001.pdf]

Supporting Information

Morphological Control of Single-Concave Elastomeric Colloid through Cross-Linking and Osmotic Pressure Variations for Chemical Delivery

*Yi-Chen Ho[1†], Ting-Yu Xu[1†], Chieh-Yun Juan[1], Yi-Shan Lai[1], Yu-Fang Lai[1], Pei-Chieh Tseng[1], and Han-Yu Hsueh[1,2]**

†Yi-Chen Ho and Ting-Yu Xu contributed equally to this work as first authors.

[*] Prof. Dr. H.-Y. Hsueh
Department of Material Science and Engineering, National Chung Hsing University, Taichung 40227, Taiwan, Republic of China.
Innovation and Development Center of Sustainable Agriculture (IDCSA), National Chung Hsing University, Taichung 40227, Taiwan, Republic of China.
E-mail: hyhsueh@nchu.edu.tw

Ms. Y.-C. Ho, Ms. T.-Y. Xu, Ms. C.-Y. Juan, Ms. Y.-S. Lai, Ms. Y.-F. Lai, Ms. P.-C. Tseng
Department of Material Science and Engineering, National Chung Hsing University, Taichung 40227, Taiwan, Republic of China.

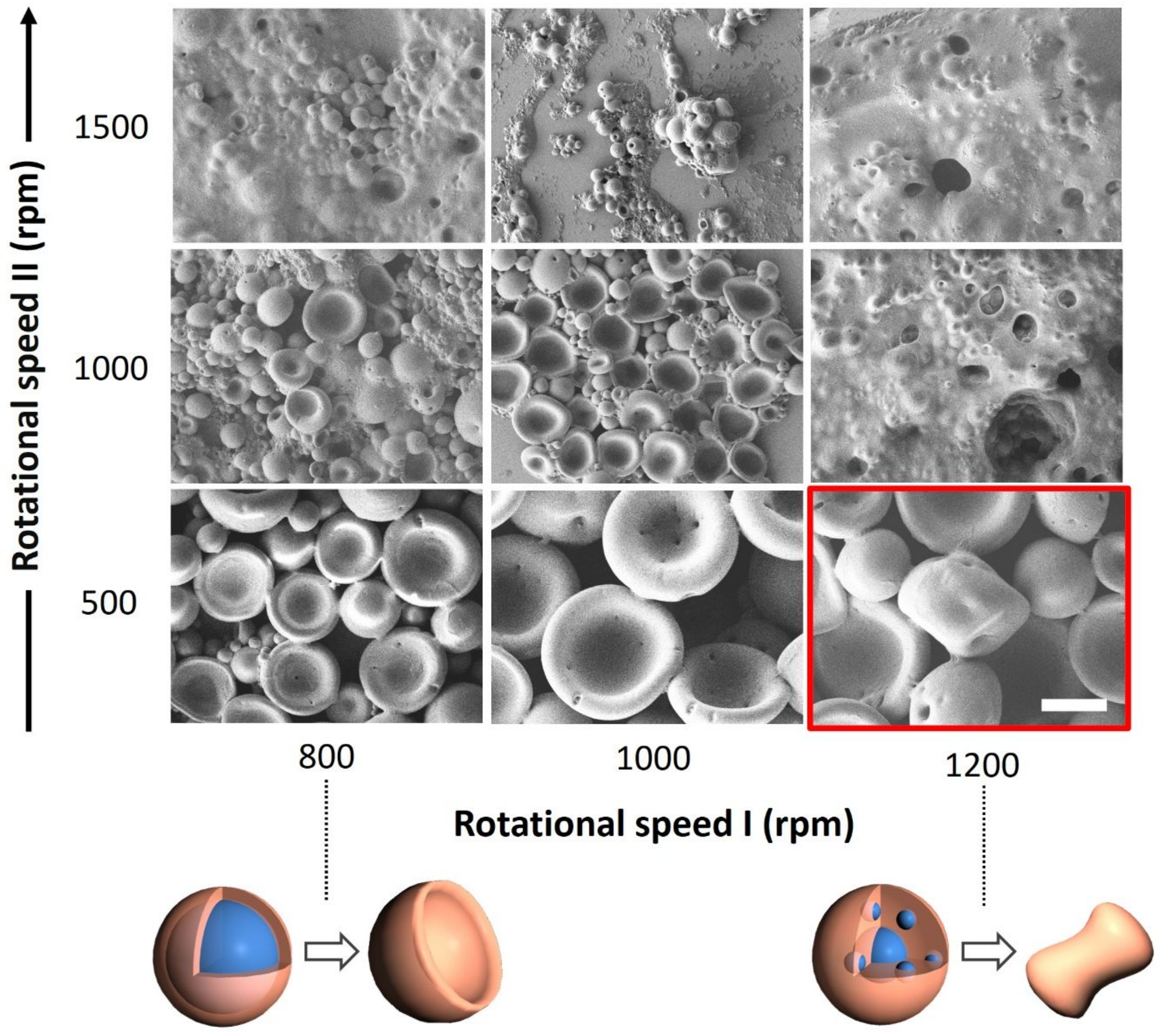

Figure S1. SEM images of PDMS micellar structures fabricated under different rotational speeds (rotational speed ranges I and II). The image highlighted by red lines shows polyhedral ridge structures. The scale bar represents 100 μm.

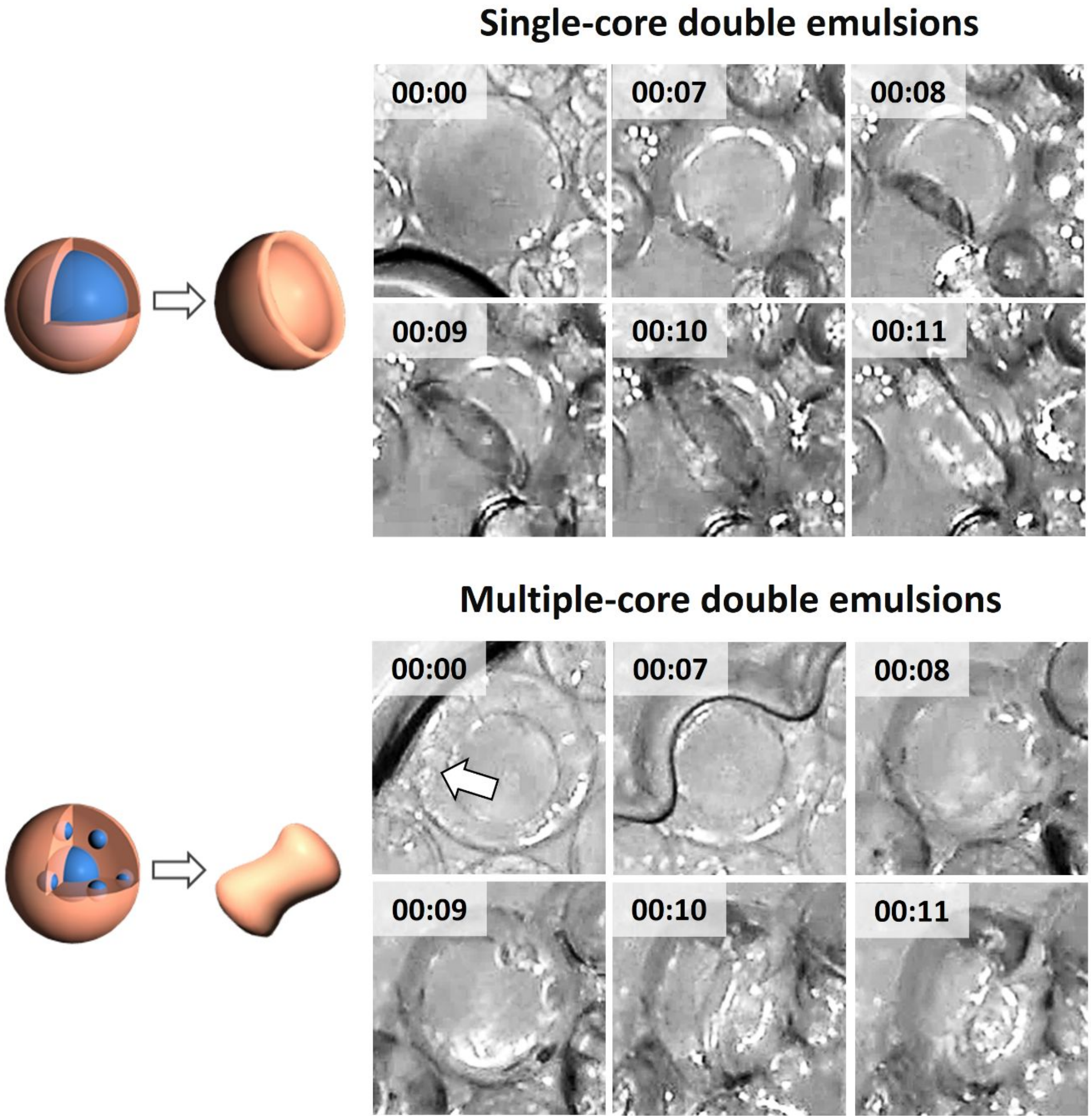

Figure S2. Images extracted from a video showing the formation of buckled PDMS shells (top section) and polyhedral ridge structures (bottom section) from single-core and multicore double-emulsion solutions, respectively. The white arrow indicates numerous smaller spheres generated under rotational speed range I.

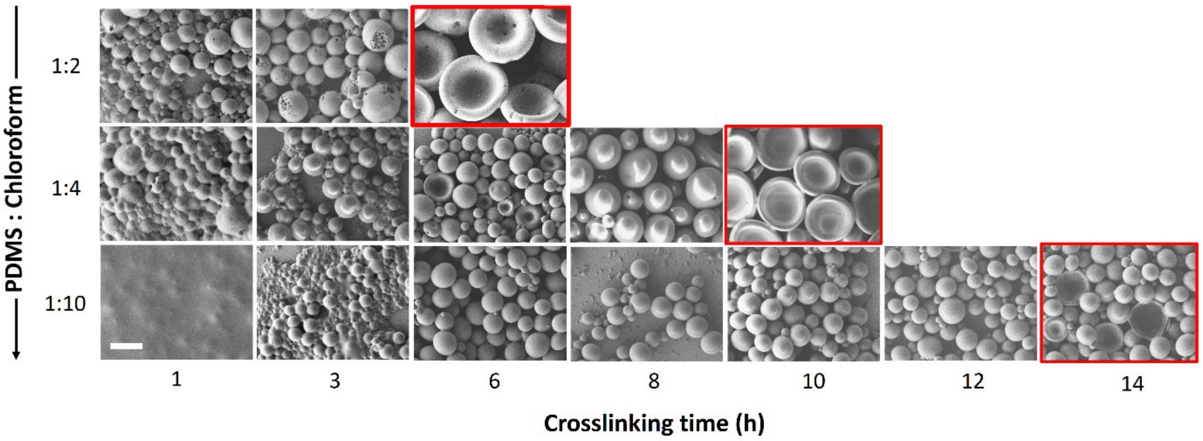

Figure S3. SEM images of PDMS micellar structures fabricated using PDMS–chloroform solutions with different mixing ratios and cross-linking times. The scale bar represents 100 μm.

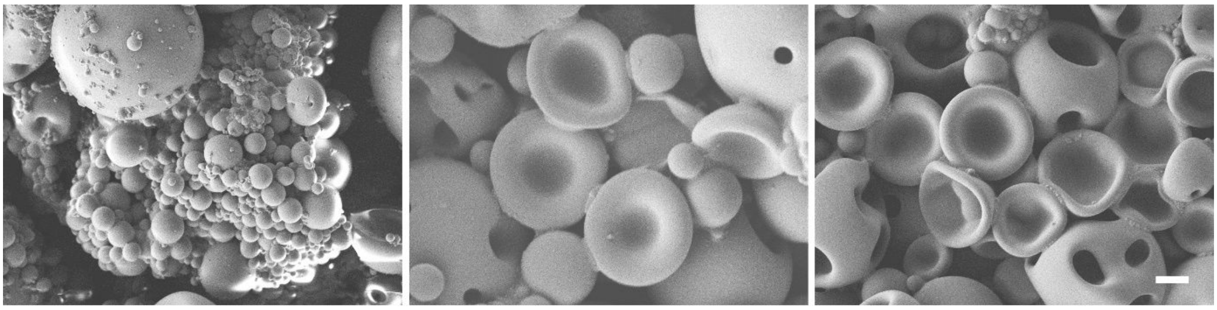

Figure S4. SEM images of buckled spherical epoxy structures fabricated from PVA solutions (W_1) with different concentrations. The epoxy resin:chloroform solvent ratio was 1:2, and the W_1 concentration ranged from 1 to 5 wt %. The scale bar represents 10 µm.

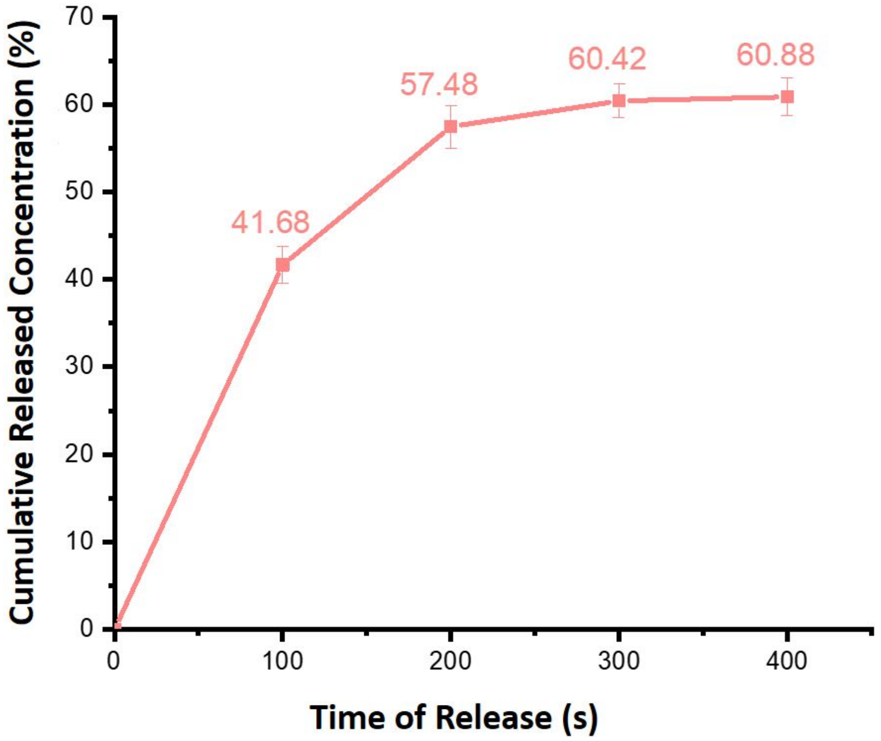

Figure S5. Percent cumulative concentration of Nile Red dye released from PDMS microcapsules into the ethanol surroundings over time at room temperature.

Table S1. Summary of the achieved internal aqueous phase (W_1), initial particle diameter (*d*), post-deformation particle diameter (*h*), and asymmetric ratio (*d*/*h*) under various PDMS-to-chloroform ratios.

PDMS: Chloroform	W_1 concentration (%)	*d* (μm)	*h* (μm)	Asymmetric ratio (*d*/*h*)
1:2	0	108.47±10.86	108.43±11.07	1.00±0.01
	1	121.07±11.67	80.28±2.70	1.51±0.14
	3	160.86±12.91	91.04±6.69	1.77±0.17
	5	203.48±16.75	103.00±9.28	1.98±0.15
1:4	0	91.35±5.72	91.14±5.44	1.00±0.01
	1	82.69±7.28	70.85±9.39	1.18±0.14
	3	54.64±6.21	38.61±4.31	1.42±0.07
	5	52.10±4.85	34.70±5.10	1.51±0.14
1:10	0	61.88±5.98	61.82±5.39	1.00±0.01
	1	47.91±9.17	40.31±7.03	1.19±0.08
	3	69.74±10.83	64.20±10.15	1.09±0.08
	5	39.48±4.39	39.37±4.46	1.00±0.00
